# Mitigation potential of selenium nanoparticles and riboflavin against arsenic and elevated temperature stress in *Pangasianodon hypophthalmus*

**DOI:** 10.1038/s41598-020-74911-2

**Published:** 2020-10-21

**Authors:** Neeraj Kumar, Sanjay Kumar Gupta, Nitish Kumar Chandan, Shashi Bhushan, Dilip Kumar Singh, Paritosh Kumar, Prem Kumar, Goraksha C. Wakchaure, Narendra Pratap Singh

**Affiliations:** 1 ICAR-National Institute of Abiotic Stress Management, Malegaon, Baramati, Pune, Maharashtra 413115 India; 2 ICAR-Indian Institute of Agricultural Biotechnology, Ranchi, Jharkhand 834010 India; 3 ICAR-Central Institute of Freshwater Aquaculture, Bhubaneswar, Odisha 751002 India; 4 ICAR-Central Institute of Fisheries Education, Mumbai, Maharashtra 400061 India; 5 ICAR-Central Institute of Brackishwater Aquaculture, Chennai, Tamil Nadu 600028 India

**Keywords:** Zoology, Animal physiology, Ichthyology

## Abstract

Climate change impact has disturbed the rainfall pattern worsening the problems of water availability in the aquatic ecosystem of India and other parts of the world. Arsenic pollution, mainly through excessive use of groundwater and other anthropogenic activities, is aggravating in many parts of the world, particularly in South Asia. We evaluated the efficacy of selenium nanoparticles (Se-NPs) and riboflavin (RF) to ameliorate the adverse impacts of elevated temperature and arsenic pollution on growth, anti-oxidative status and immuno-modulation in *Pangasianodon hypophthalmus*. Se-NPs were synthesized using fish gill employing green synthesis method. Four diets i.e., Se-NPs (0 mg kg^−1^) + RF (0 mg kg^−1^); Se-NPs (0.5 mg kg^−1^) + RF (5 mg kg^−1^); Se-NPs (0.5 mg kg^−1^) + RF (10 mg kg^−1^); and Se-NPs (0.5 mg kg^−1^) + RF (15 mg kg^−1^) were given in triplicate in a completely randomized block design. The fish were treated in arsenic (1/10th of LC_50_, 2.68 mg L^−1^) and high temperature (34 °C). Supplementation of the Se-NPs and RF in the diets significantly (p < 0.01) enhanced growth performance (weight gain, feed efficiency ratio, protein efficiency ratio, and specific growth rate), anti-oxidative status and immunity of the fish. Nitroblue tetrazolium (NBT), total immunoglobulin, myeloperoxidase and globulin enhanced (p < 0.01) with supplementation (Se-NPs + RF) whereas, albumin and albumin globulin (A:G) ratio (p < 0.01) reduced. Stress biomarkers such as lipid peroxidation in the liver, gill and kidney, blood glucose, heat shock protein 70 in gill and liver as well as serum cortisol reduced (p < 0.01) with supplementation of Se-NPs and RF, whereas, acetylcholine esterase and vitamin C level in both brain and muscle significantly enhanced (p < 0.01) in compared to control and stressors group (As + T) fed with control diet. The fish were treated with pathogenic bacteria after 90 days of experimental trial to observe cumulative mortality and relative survival for a week. The arsenic concentration in experimental water and bioaccumulation in fish tissues was also determined, which indicated that supplementation of Se-NPs and RF significantly reduced (p < 0.01) bioaccumulation. The study concluded that a combination of Se-NPs and RF has the potential to mitigate the stresses of high temperature and As pollution in *P. hypophthalmus*.

## Introduction

Climate change and pollution are major threatening factors for the growth of aquatic animals, including fishes. The altered temperature and hazardous metals disturb the homeostasis of aquatic animals leading to reduced growth, compromised immunity^1,2^, decreased anti-oxidative status and pathogenic resistance^1^ posing a serious threat to their early stages of the life of fishes^3,4^. Climate change would have a pronounced and devastating impact on fish stocks, which belong to poikilothermic animal^5,6^. Generally, the metabolic rate of fishes increases with a rise in temperature leading to the reduction of available oxygen in the water. As a result, the requirement of water flow and oxygen increases^7^ and consequently, bioaccumulation of metal (arsenic for example) in different parts of fish tissue intensifies. Reduced dissolved oxygen concentration mediated through enhanced temperature^2^ pose a stressful situation during the aerobic metabolism of the fish^6^. The effect of high temperature and metal contamination significantly reduce the ability of fish to tolerate the pollution load of the environment^8–11^.

Arsenic (As) is one of the most dangerous and hazardous metals, which adversely affects aquatic ecosystems^12^. The ubiquitous presence of As is attributed to its peculiar characteristic of origination viz. natural as well as anthropogenic sources^13^. In natural water bodies its concentration may go up to several thousand micrograms per liter^14^. It is also a dangerous carcinogenic agent, which is mainly present in the North East India such as Tripura, Mizoram, Arunachal Pradesh, West Bengal, Bihar, Jharkhand, Utter Pradesh, Haryana, Punjab and other parts of the India^15^. Arsenic was reported in major Indian rivers that encompasses groundwater of Ganga basin (4730 µg L^−1^)^16^, Ganga (4.2 µg L^−1^), Mahanadi (0.1–3 µg L^−1^), Bhagirathi-Hooghly (0.3–4 µg L^−1^)^17^ and Yamuna (32–64 µg L^−1^)^18^. Generally, there are two forms of As i.e., arsenite and arsenate, which are more toxic than methylated forms such as methylarsonate and dimethylarsinate^19^. Toxicity of As affects the physio-metabolic response, anti-oxidative status and immunity of the fishes^10^. The mode of action of As is similar to phosphate, which can replace the former in energy transfer phosphorylation reactions, leading to the impairment of ATP synthesis^19^.

Minimizing the simultaneous impacts of pollution, thermal stress and pathogenic infection for enhancement of growth performance and modulation of the immune system in culturable fish is a major challenge. Nutritional intervention could play a pivotal role in minimizing such inimical impacts on fish^8^. Riboflavin (RF) and selenium nanoparticles (Se-NPs) have been used to reduce the effects of multiple stressors (arsenic pollution and elevated temperature) and enhance growth performance and immunity of the fishes^20^. Selenium (Se) has the potential to improve anti-oxidative status and immunity of fish^21^. It is essential for activation, proliferation and differentiation of cells that control innate and adaptive immunity in humans and animals^22,23^. Riboflavin is an important nutrient and is essential for flavoprotein as a catalyst in fish. The flavin mononucleotide (FMN) and flavin adenine dinucleotide (FAD) are two active forms of riboflavin that play a crucial role in various oxidation and reduction reactions^24^.

In general, during multiple stresses, the aquatic organisms make advancement in their anti-oxidative defense system to cope up with reactive oxygen species (ROS)^25,26^. The anti-oxidative systems have both enzymatic as superoxide dismutase (SOD), catalase (CAT) and glutathione peroxidase (GPx) and non-enzymatic component such as glutathione-s-transferase (GST). SOD, CAT, GPX and GST have a major role in detoxification of ROS^8^. The low molecular weight non-enzymatic components such as antioxidants (GST) protect the cell through quenching of oxyradicals by its sulfhydryl group against oxidative stress^27^. Similarly, Se has diverse functions in antioxidant defense systems and acts as a strong antioxidant agent to protect cell membranes and other cellular components against oxidative damage^28^. Selenium also possesses defence function for biomembranes and other several cellular components from oxidative damages through reduction of a variety of hydroperoxides (ROOH), using glutathione (GSH)^29^. Riboflavin and Se-NPs have a strong ability to enhance the immune system concerning total protein, albumin, globulin, A:G ratio, blood glucose, total immunoglobulin and myeloperoxidase^22,30^. It has a major role in the regulation of the immune system of the fish against multiple stressors and pathogenic infection^31^. It also has a significant role in maintaining chaperone protein (heat shock protein) and vitamin C for the mitigation of multiple stressors. The present experiment was carried out to evaluate the mitigation potential of dietary Se-NPs and RF against arsenic and high temperature stresses in *Pangasianodon hypophthalmus*, a commercially an important aquaculture species in India.

## Material and methods

### Experimental fish and ethics statement

*Pangasianodon hypophthalmus* fingerlings (average weight, 5.35 ± 1.02 g) were obtained from Kolkata, West Bengal, India and transported to the central wet laboratory of ICAR-National Institute of Abiotic Stress Management, Baramati, Pune in healthy condition. The fish were quarantine with 5 g L^−1^ salt solution and then followed with 2 mg L^−1^ KMnO_4_ solution. Subsequently, fish were acclimatized in the cemented tank for 2 months before the commencement of the experiment and fed with a practical diet (protein 30%) at the rate of 3% body weight twice a day^22^. The water quality such as dissolved oxygen, temperature, and pH was recorded daily while ammonia was recorded weekly from all the treatments. The dissolved oxygen, temperature, pH, ammonia and other water qualities parameters were determined as per the standard protocol of the American Public Health Association, APHA^32^. The ethical guidelines for care and maintenance of the fish were strictly followed as issued by the concerned agency to minimize any discomfort to the fish during handling and sampling procedure. The present study and experimental endpoints were approved by the animal ethics committee (AEC), of Indian Council of Agriculture Research-National Institute of Abiotic Stress Management, Baramati, Pune, India.

### Experimental design

The experiment was conducted for 90 days in the 24 rectangular plastic (150 L capacity) tanks with 18 fishes in each in triplicates following a completely randomized block design. The experimental set up consisting of 8 treatment groups were as follows, control diet with no stressors (control), concurrent exposed to arsenic (2.68 mg L^−1^) and high temperature (34 °C) (As + T) and fed with control diet, groups fed with Se-NPs at the rate of 0.5 mg kg^−1^ diet and RF at the rate of 5, 10, 15 mg kg^−1^ diet with no stressors (Se-NPs + RF-5 mg kg^−1^, Se-NPs + RF-10 mg kg^−1^, and Se-NPs + RF-15 mg kg^−1^) and groups supplemented with Se-NPs at the rate of 0.5 mg kg^−1^ diet and RF at the rate of 5, 10, and 15 mg kg^−1^ diet with concurrent exposure to arsenic (2.68 mg L^−1^) and high temperature (34 °C) (Se-NPs + RF-5 mg kg^−1^ + As + T, Se-NPs + RF-10 mg kg^−1^ + As + T, and Se-NPs + RF-15 mg kg^−1^ + As + T). The manual water exchange (two-third, 66.66%) on every second day was carried out and aeration was provided with a compressed air pump throughout the experimental duration. Arsenic was added to the stressors group at the level of 1/10th of 26.8 mg L^−1^ 96 h LC_50_^10^ using sodium arsenite (NaAsO_2_) and water temperature of 34 °C maintained using the thermostatic heater.

### Preparation of fish tissue extracts using green synthesis of Se-NPs

Tissues extract was prepared using gill of *Labeo rohita*, for which the tissues were cleaned and washed in running tap water to remove blood and dust. Then the tissues were fine cut into several pieces and lysate and homogenates in a mortar pestle. The homogenates of tissues were centrifuged at 6000×*g* for 15 min at 4 °C and the final supernatant was collected for filtration using filter paper with 0.45 µm pore size to obtain the gill extract. Then the extracted gill tissues were mixed with 200 mL of sodium selenite (2 M) in distilled water and then shake for 96 h using a shaker. Then the solution was centrifuged at 6000×*g* for 15 min at 4 °C for pellet formation and then harvested and kept in the oven at 60 °C until dry, and subsequently stored at room temperature. Before mixing into the fish feed the dry pellet was crushed into fine powder^33–35^.

### Characterization of selenium nanoparticles

**Figure 1:**
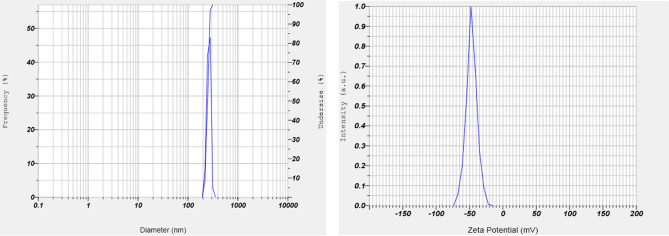
Particle size (249.4 nm) and zeta potential (− 47 mV) of selenium nano particles.

The synthesized Se-NPs was evaluated through an absorption spectrum at 300–500 nm in UV–Vis spectrophotometer (Shimadzu, UV-1800, Japan) and peak obtained in the range of 360–380 nm. The final synthesized Se-NPs were mixed with Milli-Q water and then determined particle size and zeta potential by using nano-size analyzer. The mean particle size 249.4 nm and mean zeta potential − 47 mV (Fig. 1) were obtained using Nanoparticles Analyzer (Horiba Scientific Nanoparticles Analyzer, nano partica SZ-100 series Kyoto, Japan) at 25 °C.

### Experimental diet and proximate analysis of feed

Four iso-caloric and iso-nitrogenous experimental diets were prepared. The diet containing good quality of fishmeal, groundnut meal and soybean meal as a protein source. The other ingredients added were wheat flour, sunflower oil and cod liver oil. The vitamin and mineral mixture free from selenium and riboflavin were prepared manually. Finally, the ingredient were appropriately mixed and steam cooked except vitamin and mineral mixture and Se-NPs and RF^20^ (Table 1). The proximate composition of the diet was also determined as per standard methods of AOAC^36^. The proximate composition such as protein estimation using nitrogen content, similarly ether extract measured by solvent extraction method and total carbohydrate were determined by total carbohydrate % = 100 − (CP% + EE% + Ash%). The digestible energy of the diet was determined using Halver method^37^.

**Table 1:** Ingredients composition (%) of the different experimental diets fed to *Pangasianodon hypophthalmus* during the experimental period of 90 days.

Ingredient	Control diet	Selenium nanoparticles (Se-NPs) + Riboflavin diet		
		0.5 mg kg^−1^ + 5 mg kg^−1^ diet	0.5 m g kg^−1^ + 10 mg kg^−1^ diet	0.5 mg kg^−1^ + 15 mg kg^−1^ diet
Soybean meal^a^	35.5	35.5	35.5	35.5
Fish meal^a^	20.0	20.0	20.0	20.0
Groundnut meal^a^	10.0	10.0	10.0	10.0
Wheat flour^a^	24.47	24.4645	24.4595	24.4545
Sunflower oil^a^	4.5	4.5	4.5	4.5
Cod liver oil^a^	1.5	1.5	1.5	1.5
CMC^b^	2.0	2.0	2.0	2.0
Vitamin and mineral mix^c^	2.0	2.0	2.0	2.0
Vitamin C^d^	0.03	0.03	0.03	0.03
Selenium Nanoparticles (Se-NPs)	0	0.0005	0.0005	0.0005
Riboflavin supplementation^b^	0	0.005	0.010	0.015
**Proximate analysis of experimental diets**				
CP^1^	35.51 ± 0.57	35.53 ± 0.47	34.71 ± 0.29	35.46 ± 0.35
EE^2^	10.57 ± 0.07	11.05 ± 0.28	10.99 ± 0.26	10.51 ± 0.19
Ash	9.35 ± 0.06	9.47 ± 0.14	9.73 ± 0.07	9.56 ± 0.04
TC^3^	44.57 ± 0.29	43.95 ± 0.34	44.57 ± 0.19	44.47 ± 0.59
OM^4^	90.22 ± 0.03	89.90 ± 0.48	91.52 ± 0.60	91.39 ± 1.22
DM^5^	92.66 ± 0.50	91.99 ± 0.30	91.65 ± 0.15	90.77 ± 0.05
DE^6^	415.48 ± 1.95	417.37 ± 3.28	416.0 ± 1.06	414.34 ± 0.56
Selenium (mg kg^−1^ diet)	0.24 ± 0.04	0.94 ± 0.10	0.91 ± 0.14	0.97 ± 0.11

Data expressed as Mean ± SE (n = 3).

Composition of vitamin mineral mix (quantity/250 g starch powder): vitamin A 55,00,00 IU; vitamin D3 11,00,00 IU; vitamin B1:20 mg; vitamin E 75 mg; vitamin K 100 mg; vitamin B12 0.6 mcg; calcium pantothenate 2,50 mg; nicotinamide 1000 mg; pyridoxine: 100 mg; Mn 2700 mg; I 100 mg; Fe 750 mg; Cu 200 mg; Co 45 mg; Ca 50 g; P 30 g.

*CP*^*1*^ crude protein, *EE*^*2*^ ether extract, *TC*^*3*^ total carbohydrate, *OM*^*4*^ organic matter, *DM*^*5*^ dry matter, *DE*^*6*^ digestible energy.

^a^Procured from local market.

^b^Himedia Ltd.

^c^Prepared manually and all components from Himedia Ltd.

^d^SD Fine Chemicals Ltd., India.

### Tissue homogenate preparation and blood collection

The fish tissues such as muscle, gill, liver, brain, and kidney were dissected from anesthetized fish (clove oil, 50 µL L^−1^) under aseptic conditions. The chilled sucrose (5% w/v, 0.25 M) and EDTA solution (1 mM) were used as homogenates for tissue homogenization using a homogenizer (Omni Tissue Master Homogenize, Kennesaw, GA). At the time of homogenization, the tube containing the sample was kept on ice to avoid denaturation of the enzymes by overheating. Then, the homogenates samples were centrifuged at 5000×*g* for 15 min at 4 °C in a cooling centrifuge (Eppendorf AG, 5430R, Hamburg, Germany), and further supernatants were collected and stored at − 20 °C until further analysis. During dissection, the blood was also collected from the same fish of each tank and serum was processed from the same collected blood^38^. Lowry protein assay^39^ was used for tissue protein analysis.

### Sample preparation for analysis of arsenic and selenium

Different fish tissues such as liver, muscle, gill, kidney and brain were collected as described above for arsenic and selenium analysis. The tissues were digested in an acidic condition in the microwave digestion system (Microwave Digestion System, Model START-D, SN-135177, Milestone, USA). The HNO_3_ and H_2_O_2_ in 5:1 were used for acidic digestion. The filtration of the digested samples was accomplished using Whatman paper (pore size-0.45 µm). Then the volume of digested solution was made up to 50 mL to proceed for selenium and arsenic analysis. The water samples were also collected in plastic bottles on every 15 days interval from each replicates till 90 days and stored in the refrigerator. At the time of analysis, the water samples were mixed (pooled) in triplicates in treatment wise. The different fish tissues and water samples were analyzed through inductively coupled plasma mass spectrometry (ICP-MS). (Agilent 7700 series, Agilent Technologies, USA). Multi-element Calibration Standard (Agilent Technologies, USA) solutions of 10 µg mL^−1^ was used to prepare the calibration curve. The calibration curves with R^2^ > 0.999 were accepted for concentration calculation^40^.

### Growth performance study

The fish were sampled for growth performance on every 15 days interval till 90th days. The growth performance were determined in terms of final weight gain (%) (FWG %), feed efficiency ratio (FER), protein efficiency ratio (PER) and specific growth rate (SGR) as our previous work followed this method^34^.

$${\text{Final weight gain}}\left( \% \right) \, = {\text{Final body weight}}\left( {\text{FBW}} \right) - {\text{initial body weight }}\left( {\text{IBW}} \right)/{\text{initial body weight }}\left( {\text{IBW}} \right) \, \times 100/$$

$${\text{FER }} = {\text{ Wet weight gain }}\left( {\text{g}} \right)/{\text{total dry feed intake }}\left( {\text{g}} \right)/$$

$${\text{SGR }} = { 1}00 \, \left( {{\text{ln FBW}} - {\text{ln IBW}}} \right)/{\text{number of days}}$$

$${\text{PER}} = {\text{ Total wet weight gain }}\left( {\text{g}} \right)/{\text{crude protein intake }}\left( {\text{g}} \right)$$

### Antioxidant enzyme activities

Superoxide dismutase (SOD) (EC 1.15.1.1) activities in different fish tissues were determined by Misra and Fridovich^41^. Catalase (EC 1.11.1.6) was determined as followed as a procedure of Takahara et al.^42^. The glutathione S-transferase (GST) (EC 2.5.1.18) was determined as per the procedure of Habing et al.^43^. Glutathione peroxidase (GPx) (EC 1.11.1.9) activity was accomplished following the method of Paglia and Valentine^44^.

### Lipid peroxidation (LPO)

Lipid peroxidation was determined in different fish tissues as per method followed of Uchiyama and Mihara^45^. Briefly, 0.25 mL of sample homogenates were mixed with10 mM butylated hydroxytoluene (BHT). Then 1% of phosphoric acid were mixed with 0.67% of thiobarbituric acid (TBA) and incubated at 90 °C for 45 min. the final reading was obtained at 535 nm.

### Neurotransmitter enzyme activities

The acetylcholine esterase (AChE; EC 3.1.1.7) activities were determined as followed by Hestrin et al.^46^. The final reading was obtained at 540 nm.

### Cortisol and HSP-70

Serum cortisol and HSP 70 were determined using ELISA kit (Commercially available Cortisol EIA kit, catalogue no. 500360, Cayman Chemicals, USA). Similarly, HSP 70 was also determined through EIA kit (catalogue no. EKS-700B, Bioguenix/Enzo Life Science, Mumbai, India). The assay was performed as per instruction provided with the kit. The final reading was obtained at 420 nm using ELISA plate reader (Clario Star, BMG Labtech, Germany).

### Ascorbic acid (vitamin C)

Ascorbic acid was estimated from brain and muscle tissue, followed by the method of Roe and Keuther^47^.

### Nitroblue tetrazolium (NBT), serum protein and A:G ratio

NBT activities determined as followed as Secombes^48^ and modified by Stasiack and Baumann^49^. The serum protein was estimated by using a protein estimation kit. Albumin was estimated by method of Doumas et al.^50^ and globulin was quantified by subtracting albumin values from total plasma protein.

### Myeloperoxidase content and total immunoglobulin level

The myeloperoxidase was quantified as method of Quade and Roth^51^ with some modifications^52^ and total immunoglobulin level was determined as method of Anderson and Siwicki^53^.

### Blood glucose

The determination of blood glucose was determined as per the method of Nelson^54^ and Somoyogi^55^. The final reading was obtained at 540 nm against the blank.

### Challenge study with Aeromonas hydrophila

After 90 days of the feeding trial, 8 fishes per replicates in each treatment were challenged with virulent *A. hydrophilla* (Lot no. 637-51-5 and Ref 0637P, HiMedia, Mumbai). *A. hydrophilla* was grown on a nutrient broth for 24 h at 37 °C in a BOD incubator and harvested by centrifuging the culture broth at 10,000×*g* for 10 min at 4 °C. The cells were then washed thrice in sterile PBS (pH 7.2) and the final concentration was maintained at 10^8^ CFU mL^−1^. The fishes were intraperitoneally injected with 0.15 mL of bacterial suspension in each treatment group. The fish mortality in each treatment group was recorded up to 7 days of challenge study. The tissues were dissected out from morbid fish for confirmation of *A. hydrophilla* as a causative agent for death. This method was used in our previous works^22,34^.

$${\text{Cumulative mortality }}\left( \% \right) \, = \, \frac{{\text{Total mortality in each treatment after challenge}}}{{{\text{Total no}}.{\text{ of fish challenged for the same treatments}}}} \times 100$$

$${\text{Relative }}\% {\text{ survival }} = \, \frac{{{\text{Mortality }}\left( \% \right){\text{ control}} - {\text{mortality }}\left( \% \right){\text{ treatment}}}}{{{\text{Mortality }}\left( \% \right){\text{ control}}}} \times 100$$

### Statistics

Group of the tanks were used as the experimental units for data on growth, while distinct fish were used as the experimental units for data on biochemical parameters, immune parameters, and stress biomarkers, as no tank specific response effect was noticed during the experimental trial. Data were analysed using Statistical Package for the Social Sciences program version 16.0 (SPSS Inc., Chicago, IL, USA). The data were expressed as mean ± standard error of mean and tested for normality and homogeneity of variance using the Shapiro–Wilk’s and Levene’s test, respectively. When both tests were satisfied, an ordinary one-way ANOVA (Analysis of variance) with Duncan’s multiple range tests (DMRT) was employed to test the statistical significant difference at p < 0.05, where the diet was used as an explanatory variable.

## Results

### Concurrent exposure to arsenic and temperature elicits primary stress response (cortisol) but dietary supplementation of Se-NPs and RF counteract it

**Figure 2:**
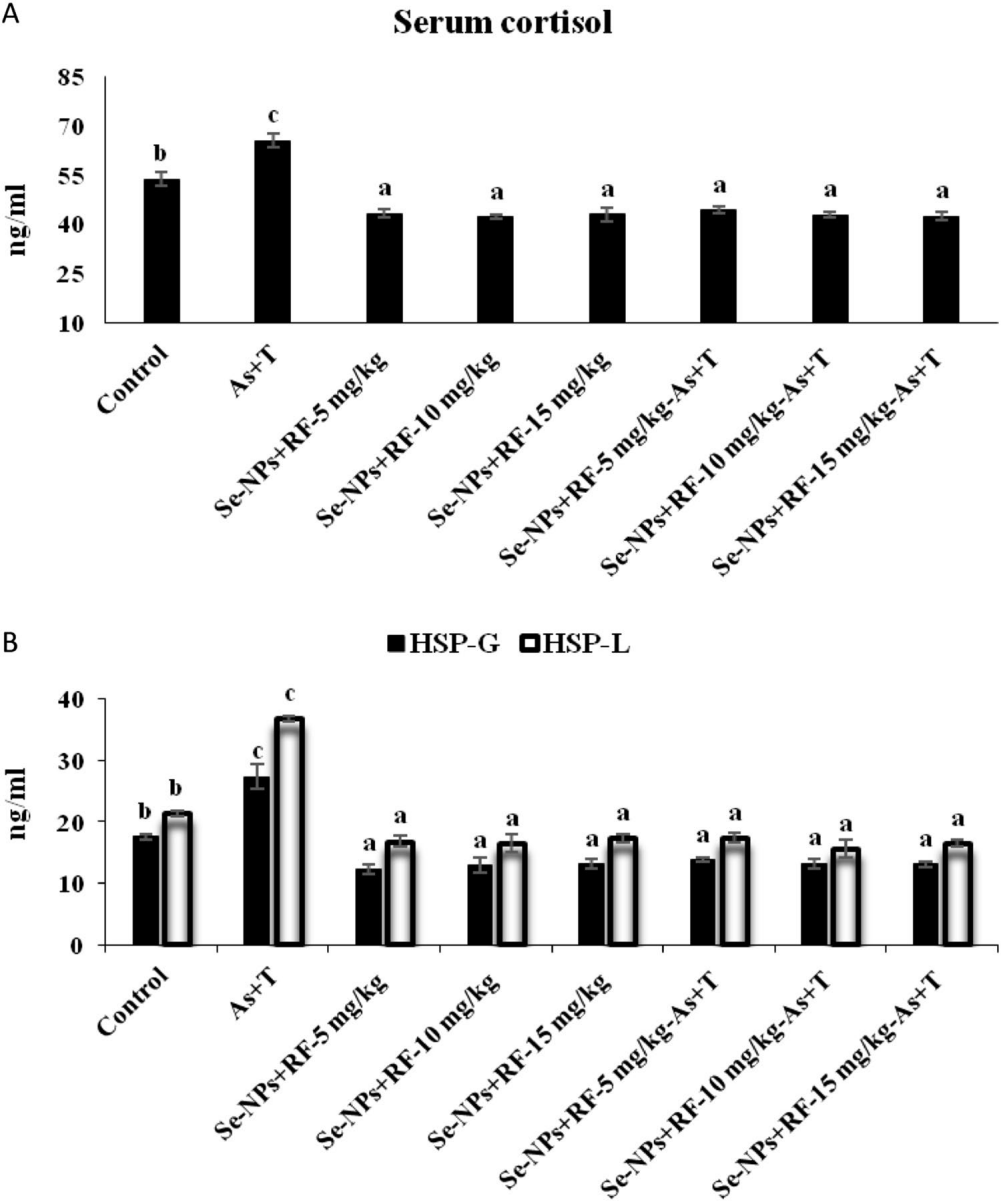
(**A**,**B**) Effect of dietary selenium nanoparticles and riboflavin on cortisol and HSP 70 of *P. hypophthalmus* reared under arsenic and high temperature for 90 days. Within endpoints and groups, bars with different superscripts differ significantly (a–c) (p < 0.01). Data expressed as Mean ± SE (n = 3).

The primary stress response, such as cortisol is the immediate effect on the hormonal system concerning multiple stresses (arsenic and temperature). The primary stress response was quantified in the form of cortisol. The cortisol level was noticeably increased (p < 0.01) with concurrent exposure to arsenic and temperature. However, the supplemented dietary Se-NPs and RF prevented the effects of multiple stresses and reduced cortisol level (Fig. 2A) in comparison to the control group and concurrent exposure to arsenic and temperature and fed with the control diet group.

### Concurrent exposure to arsenic and temperature elicits secondary stress response but dietary supplementation of Se-NPs and RF counteract it

The secondary stress response in terms of anti-oxidative status (CAT, SOD, GST and GPx) and LPO has been shown in Tables 2, 3 and 4. Concurrent exposure to arsenic and high temperature significantly enhanced (p < 0.01) the cellular stress indicators (CAT, SOD, GST and GPX) in the liver, gill, brain and kidney except SOD in the brain and kidney. The supplementation of Se-NPs and RF concurrently exposed to multiple stressors (arsenic and temperature) led to insignificant change (p > 0.05) of SOD activities in the brain and kidney (Table 2). The supplementation of dietary Se-NPs at the rate of 0.5 mg kg^−1^ diet and RF at the rate of 5, 10 and 15 mg kg^−1^ significantly (p < 0.01) reduced the impact of multiple stressors (As + T) in terms of CAT, GST, GPx and gill SOD except liver GST in comparison to unexposed (control group) and stressors group (As + T) fed with the control diet. In case of phase II enzymes (GST) in the liver, Se-NPs at the rate of 0.5 mg kg^−1^ diet and RF at the rate of 10 mg kg^−1^ diet led to noticeably (p < 0.01) protection of the tissue more prominently (p < 0.01) in compared to RF at the rate of 5 and 15 mg kg^−1^ diet concurrently exposed to multiple stressors (Table 3). Similarly, the heat shock protein (HSP 70) was significantly elevated (p < 0.01) with concurrent exposure to arsenic and temperature in gill and liver tissue. While supplementation of dietary Se-NPs at the rate of 0.5 mg kg^−1^ diet and RF at the rate of 5, 10, 15 mg kg^−1^ diet significantly reduced (p < 0.01) the HSP 70 level from multiple stressors (arsenic and temperature) (Fig. 2B) in comparison to unexposed (control group) and stressors group (As + T). In case of LPO, the activities in the liver, gill, kidney and brain have been noticeably enhanced (p < 0.01) with concurrent exposure to arsenic and high temperature. Whereas, application of Se-NPs at the rate of 0.5 mg kg^−1^ diet and RF at the rate of 5, 10 and 15 mg kg^−1^ diet significantly (p < 0.01) reduced the level of LPO in the liver, gill and kidney in compared to unexposed (control group) and stressors group (As + T). While, the brain LPO was significantly (p < 0.01) reduced in the non-stressors group fed with Se-NPs at the rate of 0.5 mg kg^−1^ diet and RF at the rate of 10 mg kg^−1^ diet in compared to control and stressors group (As + T) (Table 4).

**Table 2:** Effect of dietary selenium nanoparticles and riboflavin on catalase (CAT) and superoxide dismutase (SOD) in liver, gill, brain and kidney tissues of *P. hypophthalmus* reared under arsenic and high temperature for 90 days.

Treatments	Non-stressors	Stressors (arsenic + temperature)	Non-stressors			Stressors (arsenic + temperature)		
Diets	Control	Control	Se-NPs + RF-5 mg kg^−1^	Se-NPs + RF-10 mg kg^−1^	Se-NPs + RF-15 mg kg^−1^	Se-NPs + RF-5 mg kg^−1^	Se-NPs + RF-10 mg kg^−1^	Se-NPs + RF-15 mg kg^−1^
CAT-liver	6.00^b^ ± 0.30	15.29^c^ ± 0.60	1.50^a^ ± 0.16	1.43^a^ ± 0.20	1.40^a^ ± 0.10	1.45^a^ ± 0.17	1.56^a^ ± 0.11	1.60^a^ ± 0.16
CAT-gill	6.68^b^ ± 0.30	14.27^c^ ± 1.76	2.43^a^ ± 0.26	2.68^a^ ± 0.24	2.38^a^ ± 0.30	2.43^a^ ± 0.35	2.43^a^ ± 0.29	2.47^a^ ± 0.35
CAT-brain	3.33^b^ ± 0.22	6.92^c^ ± 0.75	1.51^a^ ± 0.19	1.66^a^ ± 0.47	1.68^a^ ± 0.26	1.79^a^ ± 0.30	1.51^a^ ± 0.27	1.63^a^ ± 0.16
CAT-kidney	5.29^b^ ± 0.75	12.29^c^ ± 1.14	2.37^a^ ± 0.26	2.33^a^ ± 0.37	2.44^a^ ± 0.32	2.39^a^ ± 0.44	2.44^a^ ± 0.19	2.49^a^ ± 0.35
SOD-liver	56.57^a^ ± 2.19	60.43^b^ ± 2.30	57.24^a^ ± 1.53	58.02^a^ ± 2.55	60.20^b^ ± 1.43	58.54^b^ ± 0.93	56.52^b^ ± 1.63	58.43^b^ ± 1.15
SOD-gill	40.00^b^ ± 0.90	44.41^c^ ± 1.16	39.86^b^ ± 1.48	38.76^a^ ± 0.98	36.91^a^ ± 1.36	39.39^b^ ± 1.88	37.47^a^ ± 1.28	38.74^a^ ± 1.72
SOD-brain	38.17 ± 0.93	37.14 ± 1.10	39.31 ± 0.81	39.17 ± 0.92	39.37 ± 0.59	39.26 ± 0.62	38.29 ± 2.25	40.24 ± 0.87
SOD-kidney	36.19 ± 1.23	37.53 ± 1.39	35.05 ± 1.02	36.64 ± 1.22	36.64 ± 0.80	36.00 ± 0.98	38.24 ± 0.68	36.63 ± 1.45

Values in the same row with different superscript (a–d) differ significantly (p < 0.01). Data expressed as Mean ± SE (n = 6). Catalase and SOD: Units/mg protein.

**Table 3:** Effect of dietary selenium nanoparticles and riboflavin on glutathione-s-transferase (GST) and glutathione peroxidase (GPx) in liver, gill, brain and kidney tissues of *P. hypophthalmus* reared under arsenic and high temperature for 90 days.

Treatments	Non-stressors	Stressors (arsenic + temperature)	Non-stressors			Stressors (arsenic + temperature)		
Diets	Control	Control	Se-NPs + RF-5 mg kg^−1^	Se-NPs + RF-10 mg kg^−1^	Se-NPs + RF-15 mg kg^−1^	Se-NPs + RF-5 mg kg^−1^	Se-NPs + RF-10 mg kg^−1^	Se-NPs + RF-15 mg kg^−1^
GST-liver	0.15^b^ ± 0.01	0.21^c^ ± 0.02	0.12^a^ ± 0.01	0.11^a^ ± 0.05	0.14^b^ ± 0.01	0.14^b^ ± 0.01	0.10^a^ ± 0.02	0.13^b^ ± 0.03
GST-gill	0.20^b^ ± 0.01	0.30^c^ ± 0.01	0.14^a^ ± 0.02	0.15^a^ ± 0.01	0.16^a^ ± 0.02	0.15^a^ ± 0.03	0.14^a^ ± 0.01	0.13^a^ ± 0.02
GST-brain	0.19^b^ ± 0.02	0.53^c^ ± 0.05	0.12^a^ ± 0.01	0.12^a^ ± 0.02	0.13^a^ ± 0.01	0.11^a^ ± 0.01	0.10^a^ ± 0.02	0.12^a^ ± 0.01
GST-kidney	0.20^b^ ± 0.01	0.29^c^ ± 0.04	0.12^a^ ± 0.01	0.12^a^ ± 0.02	0.11^a^ ± 0.01	0.10^a^ ± 0.01	0.12^a^ ± 0.03	0.10^a^ ± 0.01
GPx-liver	4.49^b^ ± 0.60	8.99^c^ ± 0.53	2.13^a^ ± 0.19	2.24^a^ ± 0.28	2.28^a^ ± 0.08	1.99^a^ ± 0.38	2.07^a^ ± 0.43	2.04^a^ ± 0.12
GPx-gill	5.74^b^ ± 0.36	9.71^c^ ± 0.41	3.49^a^ ± 0.59	3.51^a^ ± 0.21	3.55^a^ ± 0.16	3.50^a^ ± 0.59	3.35^a^ ± 0.33	3.57^a^ ± 0.28
GPx-brain	3.68^b^ ± 1.20	7.90^c^ ± 0.49	3.86^a^ ± 0.33	3.36^a^ ± 0.39	3.92^a^ ± 0.37	3.84^a^ ± 0.23	3.69^a^ ± 0.71	3.34^a^ ± 0.34
GPx-kidney	4.79^b^ ± 0.42	10.23^c^ ± 1.08	2.34^a^ ± 0.47	2.20^a^ ± 0.36	2.31^a^ ± 0.40	2.39^a^ ± 0.35	2.33^a^ ± 0.26	2.60^a^ ± 0.36

Values in the same row with different superscript (a–d) differ significantly (p < 0.01).

Data expressed as Mean ± SE (n = 6). GST and GPx: Units/mg protein.

**Table 4:** Effect of dietary selenium nanoparticles and riboflavin on lipid peroxidation (LPO) in liver, gill, brain and kidney tissues of *P. hypophthalmus* reared under arsenic and high temperature for 90 days.

Treatments	Non-stressors	Stressors (arsenic + temperature)	Non-stressors			Stressors (arsenic + temperature)		
Diets	Control	Control	Se-NPs + RF-5 mg kg^−1^	Se-NPs + RF-10 mg kg^−1^	Se-NPs + RF-15 mg kg^−1^	Se-NPs + RF-5 mg kg^−1^	Se-NPs + RF-10 mg kg^−1^	Se-NPs + RF-15 mg kg^−1^
LPO-liver	18.15^b^ ± 0.50	43.51^c^ ± 1.88	13.01^a^ ± 0.56	13.97^a^ ± 0.32	13.75^a^ ± 0.91	14.54^a^ ± 0.69	14.45^a^ ± 0.49	13.94^a^ ± 0.71
LPO-gill	12.19^b^ ± 0.85	20.75^c^ ± 1.36	8.87^a^ ± 0.42	9.16^a^ ± 0.56	9.53^a^ ± 0.51	10.35^a^ ± 0.43	10.05^a^ ± 0.42	9.42^a^ ± 0.72
LPO-kidney	24.86^c^ ± 1.47	43.58^d^ ± 0.67	21.50^b^ ± 0.83	10.63^a^ ± 0.53	22.35^b^ ± 0.88	21.73^b^ ± 1.66	11.15^a^ ± 0.82	9.73^a^ ± 0.38
LPO-brain	8.64^a^ ± 0.54	17.62^e^ ± 1.09	10.30^c^ ± 1.54	7.89^a^ ± 0.48	14.28^d^ ± 0.90	11.56^c^ ± 0.71	9.70^b^ ± 0.73	13.66^d^ ± 0.67

Values in the same row with different superscript (a–d) differ significantly (p < 0.01). Data expressed as Mean ± SE (n = 6). LPO: n mole TBARS formed/h/mg protein.

### Concurrent exposure to arsenic and temperature elicits secondary stress (acetyl choline esterase and vitamin C) response but dietary supplementation of Se-NPs and RF counteract it

**Figure 3:**
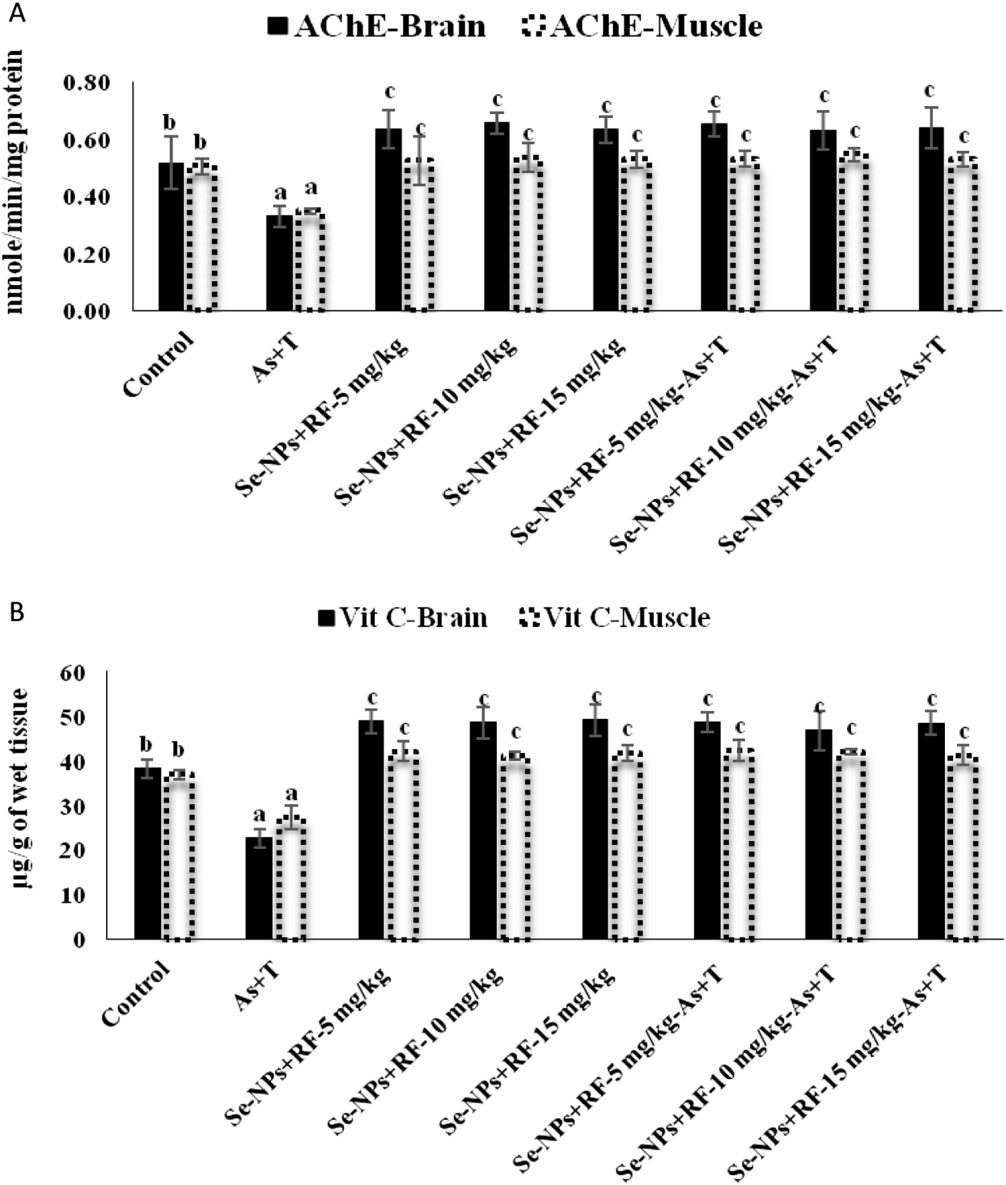
(**A**,**B**) Effect of dietary selenium nanoparticles and riboflavin on acetylcholine esterase (AChE) and vitamin C in brain and muscle within endpoints and groups of *P. hypophthalmus* reared under arsenic and high temperature for 90 days. Within endpoints and groups, bars with different superscripts differ significantly (a–c) (p < 0.01). Data expressed as Mean ± SE (n = 6).

The secondary stress response in terms of neurotransmitter (AChE) enzymes has been presented in Fig. 3A. The multiple stressors (arsenic and temperature) significantly (p < 0.01) inhibited acetylcholine esterase activities in the brain and muscle tissue. Whereas, supplementation of Se-NPs at the rate of 0.5 mg kg^−1^ diet and RF at the rate of 5, 10 and 15 mg kg^−1^ diet significantly (p < 0.01) protected acetylcholine esterase activities from inhibition and enhanced the activities in the brain and muscle tissue in compared to control group and stressor group (As + T). In the case of Vit C level in the brain and muscle, identical patterns were found as observed for AChE (Fig. 3B).

### Concurrent exposure to arsenic and temperature elicits secondary stress (immunological status) response but dietary supplementation of Se-NPs and RF counteract it

**Figure 4:**
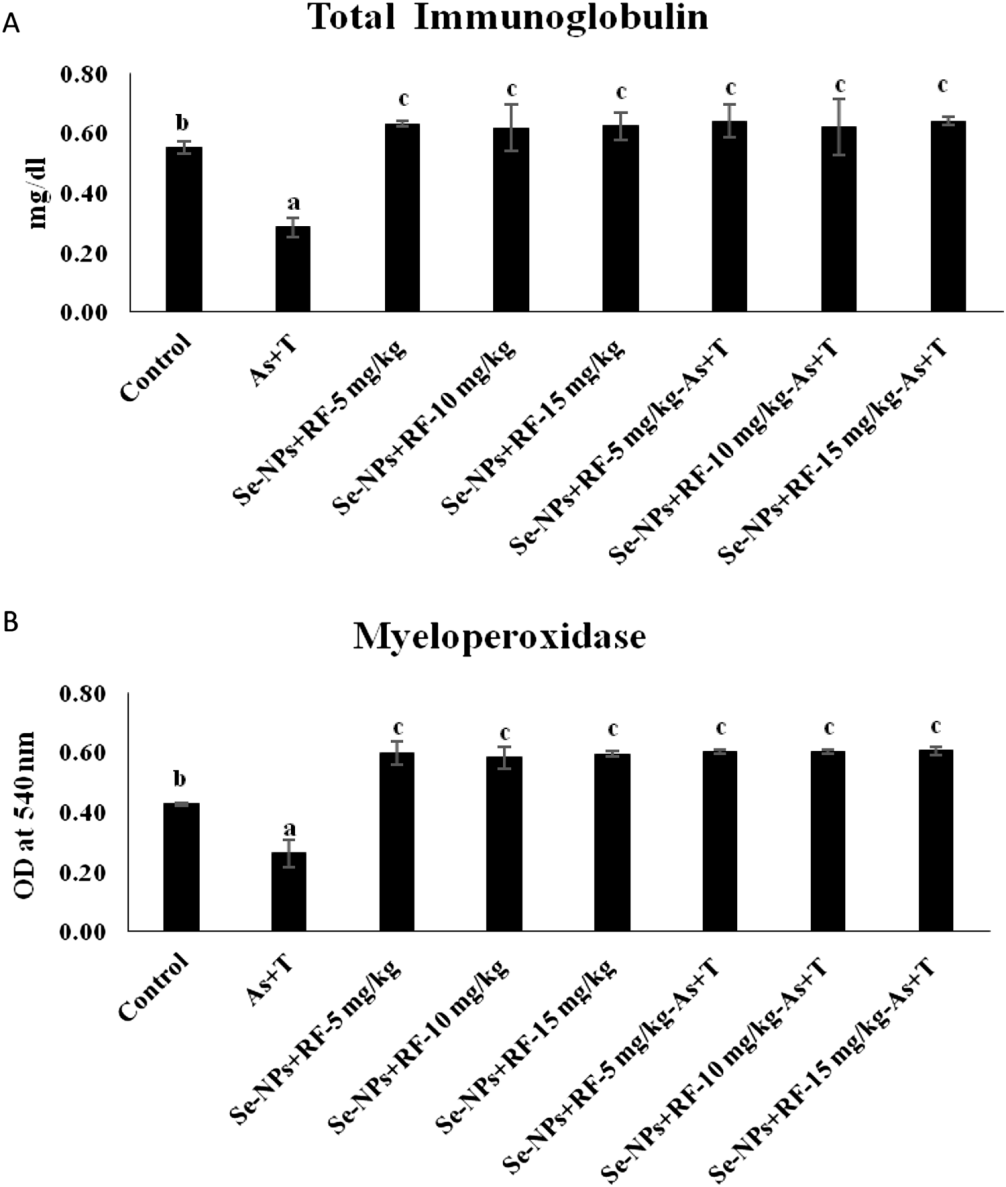
(**A**,**B**) Effect of dietary selenium nanoparticles and riboflavin on total immunoglobulin and myeloperoxidase of *P. hypophthalmus* reared under arsenic and high temperature for 90 days. Within groups, bars with different superscripts differ significantly (a–c) (p < 0.01). Data expressed as Mean ± SE (n = 3).

**Figure 5:**
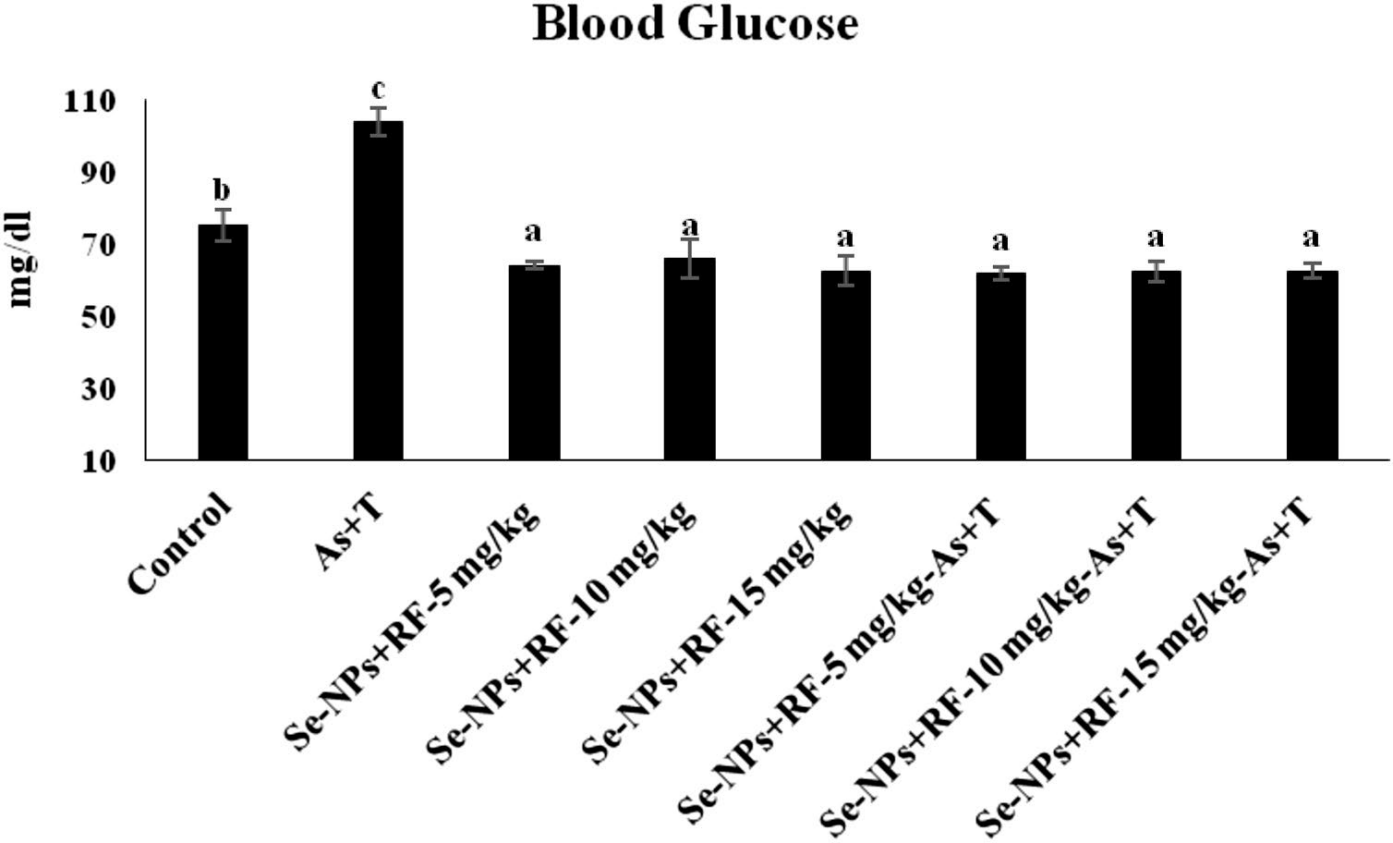
Effect of dietary selenium nanoparticles and riboflavin on blood glucose of *P. hypophthalmus* reared under arsenic and high temperature for 90 days. Within groups, bars with different superscript (a–c) differ significantly (p < 0.01). Data expressed as Mean ± SE (n = 6).

Immunological status such as total protein, albumin, A:G ratio, NBT (Table 5), total immunoglobulin, myeloperoxidase (Fig. 4A,B) and blood glucose (Fig. 5) were significantly affected (p < 0.01) with concurrent exposure to arsenic and high temperature. With exposure to multiple stressors (As + T), the total protein, globulin, NBT, total immunoglobulin and myeloperoxidase has been noticeably reduced (p < 0.01), whereas, albumin, A: G ratio and blood glucose were significantly (p < 0.01) elevated in compared to the supplemented group of Se-NPs + RF diet. Further, the application of dietary Se-NPs at the rate of 0.5 mg kg^−1^ diet and RF at the rate of 5, 10 and 15 mg kg^−1^ diet significantly (p < 0.01) enhanced the globulin, NBT, total immunoglobulin and myeloperoxidase, whereas, albumin, A: G ratio, blood glucose were significantly reduced (p < 0.01) in compared to control group and stressors group (As + T).

**Table 5:** Effect of dietary selenium nanoparticles and riboflavin on total protein, albumin, globulin, A:G ratio and nitroblue tetrazolium (NBT) of *P. hypophthalmus* reared under arsenic and high temperature for 90 days.

Treatments	Non-stressors	Stressors (arsenic + temperature)	Non-stressors			Stressors (arsenic + temperature)		
Diets	Control	Control	Se-NPs + RF-5 mg kg^−1^	Se-NPs + RF-10 mg kg^−1^	Se-NPs + RF-15 mg kg^−1^	Se-NPs + RF-5 mg kg^−1^	Se-NPs + RF-10 mg kg^−1^	Se-NPs + RF-15 mg kg^−1^
Total protein	0.78^b^ ± 0.04	0.66^a^ ± 0.03	0.87^c^ ± 0.03	0.89^c^ ± 0.02	0.84^c^ ± 0.05	0.88^c^ ± 0.06	0.85^c^ ± 0.04	0.90^c^ ± 0.02
Albumin	0.33^c^ ± 0.01	0.22^b^ ± 0.02	0.16^a^ ± 0.02	0.15^a^ ± 0.01	0.16^a^ ± 0.03	0.16^a^ ± 0.01	0.15^a^ ± 0.01	0.14^a^ ± 0.02
Globulin	0.45^a^ ± 0.02	0.44^a^ ± 0.06	0.73^b^ ± 0.04	0.74^b^ ± 0.02	0.74^b^ ± 0.03	0.73^b^ ± 0.02	0.73^b^ ± 0.04	0.75^b^ ± 0.02
A:G ratio	0.73^c^ ± 0.01	0.50^b^ ± 0.11	0.22^a^ ± 0.03	0.21^a^ ± 0.02	0.22^a^ ± 0.04	0.22^a^ ± 0.01	0.21^a^ ± 0.02	0.21^a^ ± 0.04
NBT	0.52^b^ ± 0.01	0.45^a^ ± 0.02	0.63^c^ ± 0.04	0.64^c^ ± 0.02	0.65^c^ ± 0.05	0.66^c^ ± 0.04	0.61^c^ ± 0.01	0.62^c^ ± 0.05

Values in the same row with different superscript (a–d) differ significantly (p < 0.01). Data expressed as Mean ± SE (n = 3).

### Concurrent exposure to arsenic and temperature elicits tertiary stress (growth performance, enhanced disease susceptibility and mortality, arsenic residue) responses but dietary supplementation of Se-NPs and RF counteract it

**Figure 6:**
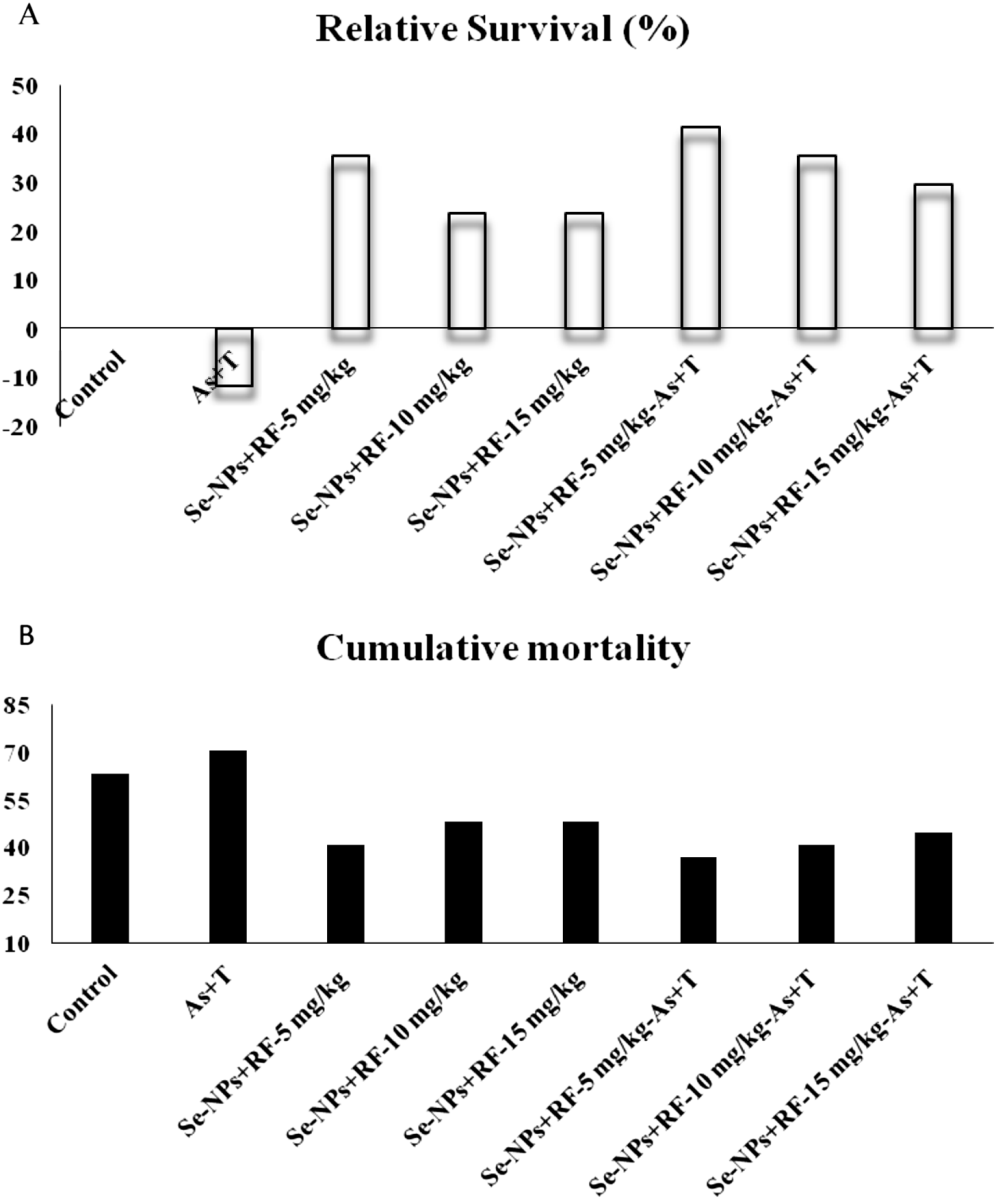
(**A**,**B**) Effect of dietary selenium nanoparticles and riboflavin on relative survival (%) and cumulative mortality of *P. hypophthalmus* after bacterial challenge reared under arsenic and high temperature for 90 days.

Table 6, summarised the results of growth performance indicators such as final weight gain (%) (FGW %), feed efficiency ratio (FER), protein efficiency ratio (PER) and specific growth rate (SGR). Final weight gain (%), feed efficiency ratio (FER), protein efficiency ratio (PER) and specific growth rate (SGR) were significantly reduced (p < 0.01) with concurrent exposure to arsenic and high temperature. Further, the application of dietary Se-NPs at the rate of 0.5 mg kg^−1^ diet and RF at the rate of 5, 10 and 15 mg kg^−1^ diet significantly (p < 0.01) enhanced the growth performance (FWG %, FER, PER and SGR) in both non-stressors and stressors condition (Table 6) compared to control and stressors group (As + T). The pathogenic infection was administrated to fish after 90 days of the experiment with *A. hydrophila* to determine the relative (%) survival and cumulative mortality in both controls fed multiple stressors group (As + T) and supplemented groups with non-stressors and stressors. Relative (%) survival (Fig. 6A) was observed in the range of − 11.76, 35.29, 23.53, 23.53, 41.18, 35.29 and 29.41% with respect to concurrent exposure to arsenic and temperature, the group fed with Se-NPs 0.5 mg kg^−1^ diet and RF at the rate of 5, 10 and 15 mg kg^−1^ diet with or without stressors groups. In case of cumulative mortality (%) (Fig. 6B), 62.96, 70.37, 40.74, 48.15, 48.15, 37.04, 40.74, 44.44% was noticed with respective to different treatment of stressors (As + T) and supplemented group (dietary Se-NPs + RF) in non-stressors and stressors condition. The concentration of As in the water was found higher in the stressor group fed with a control diet followed by stressor groups supplemented with 5, 15 and 10 mg kg^−1^ diet (Table 7). Parallel to this, increased level of As was observed in the liver, kidney brain and gill tissues of stressors group fed with control diet With dietary supplementation of Se-NPs + RF, the concentration of As in all the studied tissues except muscle of stressor group reduced compared to the stressor group fed with control diet (Table 7). There was no any significant difference (p > 0.05) was observed in the muscle tissues through the experimental groups. The bioaccumulation of selenium was also determined in the fish muscle tissue (Table 7), however, the concentration of Se was significantly higher (p < 0.01) in the group treated with Se-NPs at the rate of 0.5 mg kg^−1^ diet and RF at the rate of 5, 10 and 15 mg kg^−1^ diet in both non-stressors and stressors condition in compared to control group and in concurrent exposed to arsenic and high temperature and fed with the control diet group. The minimum concentration was determined in the group treated with arsenic and temperature and fed with control diet in comparison to all other treatments.

**Table 6:** Effect of dietary selenium nanoparticles and riboflavin on final weight gain%, FER, PER and SGR of *P. hypophthalmus* reared under arsenic and high temperature for 90 days.

Treatments	Non-stressors	Stressors (arsenic + temperature)	Non-stressors			Stressors (arsenic + temperature)		
Diets	Control	Control	Se-NPs + RF-5 mg kg^−1^	Se-NPs + RF-10 mg kg^−1^	Se-NPs + RF-15 mg kg^−1^	Se-NPs + RF-5 mg kg^−1^	Se-NPs + RF-10 mg kg^−1^	Se-NPs + RF-15 mg kg^−1^
Final weight gain %	139.78^b^ ± 11.34	82.01^a^ ± 4.77	245.34^c^ ± 8.36	249.76^c^ ± 9.42	241.24^c^ ± 8.36	246.62^c^ ± 3.45	246.14^c^ ± 8.12	244.06^c^ ± 2.10
FER	0.39^b^ ± 0.021	0.27^a^ ± 0.009	0.54^c^ ± 0.008	0.55^c^ ± 0.015	0.53^c^ ± 0.019	0.54^c^ ± 0.005	0.54^c^ ± 0.009	0.54^c^ ± 0.011
PER	1.11^b^ ± 0.041	0.78^a^ ± 0.048	1.53^c^ ± 0.018	1.61^c^ ± 0.045	1.65^c^ ± 0.049	1.70^c^ ± 0.052	1.68^c^ ± 0.019	1.74^c^ ± 0.030
SGR	0.97^b^ ± 0.052	0.66^a^ ± 0.029	1.38^c^ ± 0.027	1.39^c^ ± 0.030	1.36^c^ ± 0.027	1.38^c^ ± 0.011	1.38^c^ ± 0.026	1.37^c^ ± 0.006

Values in the same row with different superscript (a–c) differ significantly (p < 0.01). Data expressed as Mean ± SE (n = 3).

**Table 7:** Effect of dietary selenium nanoparticles and riboflavin on concentration of arsenic in experimental water and bioaccumulation in fish tissues such as liver, muscle, gill and kidney tissues and selenium bioaccumulation in muscle tissues of *P. hypophthalmus* reared under arsenic and high temperature for 90 days.

Treatments	Non-stressors	Stressors (arsenic + temperature)	Non-stressors			Stressors (arsenic + temperature)		
Diets	Control	Control	Se-NPs + RF-5 mg kg^−1^	Se-NPs + RF-10 mg kg^−1^	Se-NPs + RF-15 mg kg^−1^	Se-NPs + RF-5 mg kg^−1^	Se-NPs + RF-10 mg kg^−1^	Se-NPs + RF-15 mg kg^−1^
Water (µg L^−1^)	11.08^a^ ± 1.44	753.83^e^ ± 24.28	7.73^a^ ± 0.43	6.20^a^ ± 0.87	8.45^a^ ± 1.06	444.33^d^ ± 33.97	200.12^b^ ± 5.41	300.02^c^ ± 58.87
Liver (mg kg^−1^)	0.11^a^ ± 0.01	1.99^b^ ± 0.08	0.08^a^ ± 0.01	0.09^a^ ± 0.02	0.10^a^ ± 0.01	0.10^a^ ± 0.03	0.07^a^ ± 0.01	0.10^a^ ± 0.01
Muscle (mg kg^−1^)	0.08 ± 0.01	0.10 ± 0.01	0.03 ± 0.001	0.23 ± 0.2	0.05 ± 0.01	0.04 ± 0.01	0.02 ± 0.01	0.08 ± 0.01
Gill (mg kg^−1^)	0.13^a^ ± 0.01	1.42^c^ ± 0.11	0.54^b^ ± 0.14	0.10^a^ ± 0.001	0.12^a^ ± 0.01	0.16^a^ ± 0.05	0.11^a^ ± 0.01	0.16^a^ ± 0.03
Kidney (mg kg^−1^)	0.20^a^ ± 0.01	1.64^b^ ± 0.15	0.15^a^ ± 0.02	0.14^a^ ± 0.001	0.14^a^ ± 0.01	0.19^a^ ± 0.02	0.17^a^ ± 0.02	0.16^a^ ± 0.03
Brain (mg kg^−1^)	0.02^a^ ± 0.002	1.16^c^ ± 0.001	0.03^a^ ± 0.04	0.06^a^ ± 0.01	0.09^a^ ± 0.01	0.08^a^ ± 0.03	0.09^a^ ± 0.01	0.08^a^ ± 0.03
Muscle-Se (mg kg^−1^)	0.59^b^ ± 0.08	0.23^a^ ± 0.03	0.96^c^ ± 0.06	0.96^c^ ± 0.07	1.05^c^ ± 0.05	0.91^c^ ± 0.05	0.95^c^ ± 0.12	1.05^c^ ± 0.05

Values in the same row with different superscript (a–c) differ significantly (p < 0.01). Data expressed as Mean ± SE (n = 4).

## Discussion

Global climate change is driven mainly by anthropogenic activities and it is expected to increase in the future with an obvious increasing load of human beings on the earth. As per IPCC^56^, the mean global temperature would be increased by 0.2 °C in the next two decades and 1.8–4.0 °C by the year 2100. The present study provides eco-physiological insight with concurrently exposed metal (low dose) and high temperature as a cause for reduced productivity and the organism’s immunity. It is also possible to adopt preventive measures and mitigation strategies that would be useful to aquaculturists. Considering the above concerns, the present paper is the first novel report on significant role of Se-NPs and RF against arsenic and high temperature exposed *P. hypophthalmus*.

The primary stress response adrenocorticotropic hormone (ACTH) is responsible for secretion of the cortisol through internal steroidogenic cells and regulated by corticotropin-releasing hormone (CRH)^57^. In response to multiple stressors (arsenic and temperature), the modifications in the endocrine secretion by over/under expression of cortisol is inevitable. Nevertheless, impaired cortisol secretion may compromise the health of the fish, due to the function of cortisol in osmoregulation, metabolism, and immunity^58^. The supplementation of dietary Se-NPs at the rate of 0.5 mg kg^−1^ diet and RF at the rate of 5, 10 and 15 mg kg^−1^ diet have significantly reduced the cortisol level which might be possible due to important role of Se in formation of glutathione peroxidase, deiodinase and thioredoxin reductase^59^. In addition, Se-NPs might be help in the stimulation of ACTH for binding with membrane receptors in the steroidogenic cell and activates the cAMP protein kinase, a second messenger pathway stimulated via steroidogenic acute regulatory protein in the mitochondria by cholesterol^60^. However, the cholesterol is transferred to pregnenolone and then to cytochrome P450 enzymes in the endoplasmic reticulum and further mitochondria transform pregnenolone to cortisol^61^. Our earlier report also inferred that supplementation of Se-NPs reduced the cortisol level in fish on exposure to lead and high temperature^22,23^. Concerning secondary stress response, CAT, SOD, GST, GPx and LPO are the key components to indicate and/or maintain the cell against the stress. The temperature enhances the arsenic toxicity in the aquatic eco-system as reported in our previous study^10^. Even though, As is known as carcinogenic agent and found in two inorganic forms, arsenite (III) and arsenate (V), it may induce the cell redox system to release/or generate reactive oxygen species. Glutathione has an important role in electron donor in the reduction of arsenate to arsenite. The other mechanism is also involved as reactive nitrogen species, which is responsible for oxidative damage associated with arsenic^62^. Generally, the metals produce free radicals in two ways such as redox active metals and without redox potential. It is involved in the thiol-containing antioxidants enzyme system^63^. The other mechanism is also involved in the production of free radicals through activation of redox sensitive via transcription factors such as AP-1, p53, and NF-κB which control the expression of protective genes and stop DNA repair and influence apoptosis, cell differentiation, and cell growth^64^. In the present study, the activities of CAT, GST and GPx were significantly higher in the combined stressor groups (As + T) fed with a control diet compared to stressor groups (As + T) fed with combinatorial mixture of Se-NP and RF. The free radical altered the protein structure, cellular damage, diseases occurrence and also disturbs the equilibrium between antioxidant level and cellular pro-oxidants^9,65^. Generally cells can detoxify contamination through the oxidation process to create equilibrium in oxidants and antioxidants from aerobic metabolism^66–68^. In the present study, the combination of dietary Se-NPs and RF has significantly reduced the oxidative stress and enhanced anti-oxidative status. It might be associated due to the role of Se-NPs and RF in anti-oxidant networks and the utilization of free radical against oxidative stress^69^. Selenium is an important component for glutathione peroxidase (GPx), which was first identified as a selenoprotein. It defends the cell against oxidative injuries/stress^70^. Apart from this, it plays important role in maintaining essential nutrients in animal and human at trace level that imparts crucial antioxidative function to selenoproteins via selenocysteine. Moreover, it is a vital component for the neutralization of adverse effects of reactive oxygen species with the help of CAT, SOD, GST and GPx^71^. Apart from Se-NPs, the RF is also a vital antioxidant that helps in several anti-oxidative enzymes such as glutathione for reducing oxidization level for reduction of oxidative stress^72,73^. It is also required for the formation of vitamin B-6, which has its own antioxidant activity in the form of pyridoxal phosphate^74^. Results of our study indicate that group concurrently exposed to arsenic and high temperature and fed with control diet significantly enhanced (p < 0.01) LPO, but supplementation of Se-NPs and RF @ 5, 10 and 15 mg kg^−1^ diet significantly reduced the LPO in the liver, gill and kidney tissues. The elevated level of LPO could be attributed due to increased formation of oxygen-free radicals and alterations in the antioxidant defense system^75^ which is revealed from the results of the present study. The possible reasons for reduced LPO level due to supplementation of Se-NPs + RF might be correlated to the vital role of Se in the protection of tissues and cells through GPx enzymatic systems^76^.

The heat shock protein (HSPs) belongs to groups of various conserved proteins like chaperones produced during stress conditions. It has a diverse function during stress conditions and essential for housekeeping and cytoprotective functions^77^ along with immune response particularly, T-cell mediated response^78,79^. In this study, the HSP 70 level in the gill and liver was enhanced significantly with multiple stressors (arsenic and temperature), but further dietary supplementation of Se-NPs and RF reduced the HSP 70 level in both organs. It may have two reasons; first, Se-NPs have a function in the delivery of signal from peptides to antigen cells and second, it may have their own function like seleno-methionine. On the other side, the RF have important role to play for confirmation and assembly of vital protein which is important for down-regulation of proinflammatory cytokines, consequently preventing post-injury metabolic dysfunction and cellular injury and death^80^ and this is the reason which might have been responsibly played by RF in the present investigation.

In this study, the acetylcholine esterase (AChE) activities were significantly inhibited with concurrent exposure to arsenic and high temperature. Further, the supplementation of dietary Se-NPs and RF improved the activities of AChE. It comes under family cholinesterases (ChEs) which is resultant of carboxylic ester and esters of choline through hydrolyzation. The hydrolyzation of the neurotransmitter acetylcholine and pseudocholinesterase or butyrylcholinesterase (BChE) through AChE, which utilizes the butyrylcholine as substrate. The occurrence of AChE is mainly prevalent in the neuromuscular junctions and cholinergic synapses in the central nervous system of the animal/fish. It hydrolyzes into acetylcholine and choline after activation of acetylcholine receptors at the postsynaptic membrane^81^. Enhanced AChE activities led due to dietary supplementation of Se-NPs and RF, indicate the role of Se-NPs in interfering with the cholinergic system as validated in our previous study^22,33^. With respect to RF, it mainly depends upon the coenzymes factor via. Flavoprotein, FMN and FAD, which are very essential for rate-limiting factors for most cellular enzymatic processes^31^. Moreover, RF is indispensable to the flow of blood in the brain and chloride plexus which is regulated by multiple homeostatic mechanisms in the brain^82^. Vitamin C is the potent anti-oxidative agent and essential for collagen synthesis^83^. It has a crucial role in the metabolism of steroids, detoxification of xenobiotics, and plays crucial role in the protection of the cell against oxidative injuries^84^. Our previous study demonstrated that, supplementation of Se-NPs enhanced Vitamin C levels in the brain and muscle tissues against multiple stressors in the fish^33^.

Total protein, albumin, globulin, A:G ratio and NBT are the reliable indicator of the innate immune system in the fish. Globulin and NBT were significantly (p < 0.01) enhanced and albumin and A:G ratio were significantly reduced (p < 0.01) with supplementation of Se-NPs + RF in both non-stressors and stressors condition in compared to control fed and concurrent exposure to multiple stressors (As + T) and control group. There is four types of globulins protein such as α_1_, α_2_, β and γ^85^. It is demonstrated that the higher level of indicators, reflects higher globulin protein, which is in agreement to our results for Se-NPs and RF treated groups with the lowest A:G ratio against multiple stressors^33^. The study conducted by Javed and Usmani^86^ demonstrated that, the A:G ratio was significantly (p < 0.01) reduced in *Channa punctatus* inhabiting in pollution effluent rich river in compared to unexposed group of fish, which might be associated to sudden increase in energy demand that fulfilled through protein synthesis. The other nutritional supplements which led to enhanced immunological status (NBT, serum total protein, A: G ratio and blood glucose) in the fishes are lecithin^8^, zinc and their nanoparticles^87^ and pyridoxine^88^. The synchronized application of dietary Se-NPs and RF improved immunity of the fish might be associated with enhanced production of B-lymphocytes that enhanced the lysozyme activity in fish^33^. The albumin is essential for transport of hormones, metal, bilirubin, vitamin and drugs. It has also important role in fat metabolism and regulates the amount of free available hormone^89^. Moreover, the gamma globulins are essential for blood immunological protein and associated with the maintenance of healthy immune systems^90^. The higher level of nitro blue tetrazolium (NBT) indicates healthy non-specific immunity in which the phagocytes act for the intracellular superoxide radicals produced by leucocytes^91^. In this study, total immunoglobulin was significantly inhibited in multiple stressors group (As + T), further, the level of total immunoglobulin was enhanced with dietary supplementation of Se-NPs and RF. The immunoglobulins are fundamental constituent that plays a vital role in the adaptive immune responses^92^. It must be emphasised that immunoglobulin has an essential role in defense mechanism through restricted dispersal of infectious agents, killing of various microbes and other pathogens, repairs of tissue damage and maintenance of the healthy state of fish and other animals^93^. Myeloperoxidase is a type of haemoprotein, used during a respiratory burst in the form of hydrogen peroxide and produces hypochlorous acid^94^. The hypochlorous acid is the potent oxidant that elicitates cytotoxic effects on mammalian and bacterial cells^95^. The supplementation of Se-NPs + RF enhanced MPO level, which might be correlated due to increased activity of neutrophils and the repairment of the damaged tissues. The activated neutrophils release O_2_ derived species (H_2_O_2_) and myeloperoxidase uses H_2_O_2_ to oxidize Cl^−^ ions to form HOCl, which is potent oxidant responsible for bacterial killing activity^96^. In this study, the total immunoglobulin level was reduced on exposure to multiple stressors (As + T), however, supplementation with dietary Se-NPs and RF improved the total immunoglobulin level in the fish. It indicates that the dietary supplements act as anti-stressor and possess immunomodulatory and protective properties in fish. Blood glucose is directly related with immunity and health status of the fish. In this study, the blood glucose was significantly enhanced with concurrent exposure to arsenic and high temperature (As + T) and fed with a control diet, however, dietary supplementation (Se-NPs + RF) reduced blood glucose level. The correctness in glucose level has some reason associated with it, such as process of excessive gluconeogenesis synthesis of glucose from non-carbohydrate source mainly protein and amino acid, and the enhancement of secretion of catecholamine^97^. Apart from the above possible reasons, RF plays essential role in the stimulation of gluconeogenesis and control mechanism of adrenal cortical^98^.

The tertiary stress response in the fish has been illustrated in terms of growth performance. The higher weight gain (%) was observed in the dietary supplemented group of Se-NPs at the rate of 0.5 mg kg^−1^ diet and RF at the rate of 5, 10 and 15 mg kg^−1^ diet with or without exposure to multiple stressors (arsenic and temperature). Generally, the metal (arsenic) enters into the fish body and then accumulated inside the different organs and usually is not removed through metabolism and becomes toxic for the animal/fish^99^. This might be a possible reason for the reduced growth observed in the group exposed to arsenic and temperature.

The exposure of metal (arsenic) contamination and elevation in water temperature adversely impact fish metabolism, growth, reproduction, immune function, and enzyme activity^100^. However, rising water temperature resulting in increased oxygen consumption and metabolic rate could be the reason for aggravated stress and decreased immunity of the fish^101^. Further, when fishes exposed to multiple stressors, the feed intake rate and metabolic rate reduced, resulting in reduced growth rate^102^. In the present study, we used dietary Se-NPs and RF to enhance growth performance, against arsenic and temperature stress. Selenium has an important role in various biological functions in enzymatic oxidation–reduction and nucleic acid metabolism. It also participated in the oxidised materials such as carotenoids and vitamin A, which is responsible for increasing in protein and water in the cells^103,104^ . In addition to this, RF have an important role in riboflavin-5 phosphate and flavin adenosine dinucleotide which play an important role in metabolism for the transfer of electrons in biological oxidation–reduction reactions involving carbohydrate, lipid and protein metabolism^105^. The previous study also demonstrated that supplementation of RF helps in improving growth performance in Jian carp^106^. The other research on Se-NPs reported on growth enhancer property in crucian carp^107^, common carp^108^, *Pangasius* species^33^. The arsenic concentration has been determined in the experimental water and different fish tissues (liver, muscle, gill, kidney and brain). The selenium bioaccumulation was also determined in the fish muscle. The arsenic bioaccumulation and high temperature effect on various meta-physiological activities and immunity of the fish as reflected in present study. The concentration of arsenic was highest in concurrent exposure to arsenic and temperature and fed with control diet and the supplemented group (Se-NPs + RF) has the lowest, which might be due to property of selenium in the absorption of arsenic^109^. The study was investigated in rice seed priming in selenium overnight and cultivated in arsenic-contaminated water and found less arsenic concentration in rice seed. In the present investigation, the selenium-containing diet has been fed to fishes for 90 days, the unutilized selenium in the diet may absorb the arsenic in supplemented diet groups (Se-NPs + RF) and higher arsenic concentration in un-supplemented group. In the case of fish tissues, the supplemented group (Se-NPs + RF) significantly reduced the bioaccumulation of the arsenic due to the ability of Se-NPs and RF to accelerate detoxification of arsenic inside the body^20^^.^ The arsenic bioaccumulation effects on various meta-physiological activities and immunity of the fish as reflected in the present study. The selenium concentration was also determined in the fish muscle. The detected value of the arsenic is meagre compared to the addition of arsenic during the experiment, which might be due to the conversion of arsenic sugar^110^.

At the end of the 90 days experiment, fish were infected with pathogenic bacteria (*Aeromonas hydrophila*) to evaluate relative survival (%) and cumulative mortality. The highest mortality was observed in the multiple stressors group (arsenic and temperature) and the lowest was observed in the supplemental group with dietary Se-NPs and RF. Lower mortality demonstrated in the group supplemented with dietary Se-NPs is in accordance with our previous report^﻿34﻿^. The protective effect of Se-NPs against pathogenic infection might be associated due to its role in immunostimulation in fish as reported in our previous study^33^. Besides, Se-NPs exhibited immunostimulation efficacy through boosted innate immune response via regulation of redox-sensitive transcription factors^111^.

## Conclusion

In totality, the present study concludes that a combination of selenium nanoparticles and riboflavin are potent nutritional supplements for reducing the impact of multiple stressors in fishes. This paper is the first novel findings to describe the significant role of Se-NPs and RF in combating multiple stressors (arsenic and temperature). In this study, we visualized the impact of arsenic and high temperature (34 °C) on growth performance, anti-oxidative status, immunity, and bacterial infection, and other cellular metabolic stress. Further, the fish with already compromised stress responses could be counteracted with dietary Se-NPs and RF that enhanced immunity, growth performance, and other body indices. Therefore, it is recommended that RF at the rate of 5 mg kg^−1^ diet with Se-NPs at the rate of 0.5 mg kg^−1^ diet is appropriate for the improvement of growth and modulation of immunity in *P. hypophthalmus*.
